# Expression and function of proton-sensing G-protein-coupled receptors in inflammatory pain

**DOI:** 10.1186/1744-8069-5-39

**Published:** 2009-07-14

**Authors:** Ying-Ju Chen, Chia-Wei Huang, Chih-Shin Lin, Wen-Han Chang, Wei-Hsin Sun

**Affiliations:** 1 Department of Life Sciences, National Central University, Jhongli, Taiwan, Republic of China; 2 Institute of Systems Biology & Bioinformatics, National Central University, Jhongli, Taiwan, Republic of China; 3 Department of Physiology and Pharmacology, University of Bristol, Bristol BS8 1TD, UK

## Abstract

**Background:**

Chronic inflammatory pain, when not effectively treated, is a costly health problem and has a harmful effect on all aspects of health-related quality of life. Despite the availability of pharmacologic treatments, chronic inflammatory pain remains inadequately treated. Understanding the nociceptive signaling pathways of such pain is therefore important in developing long-acting treatments with limited side effects. High local proton concentrations (tissue acidosis) causing direct excitation or modulation of nociceptive sensory neurons by proton-sensing receptors are responsible for pain in some inflammatory pain conditions. We previously found that all four proton-sensing G-protein-coupled receptors (GPCRs) are expressed in pain-relevant loci (dorsal root ganglia, DRG), which suggests their possible involvement in nociception, but their functions in pain remain unclear.

**Results:**

In this study, we first demonstrated differential change in expression of proton-sensing GPCRs in peripheral inflammation induced by the inflammatory agents capsaicin, carrageenan, and complete Freund's adjuvant (CFA). In particular, the expression of TDAG8, one proton-sensing GPCR, was increased 24 hours after CFA injection because of increased number of DRG neurons expressing TDAG8. The number of DRG neurons expressing both TDAG8 and transient receptor potential vanilloid 1 (TRPV1) was increased as well. Further studies revealed that TDAG8 activation sensitized the TRPV1 response to capsaicin, suggesting that TDAG8 could be involved in CFA-induced chronic inflammatory pain through regulation of TRPV1 function.

**Conclusion:**

Each subtype of the OGR1 family was expressed differently, which may reflect differences between models in duration and magnitude of hyperalgesia. Given that TDAG8 and TRPV1 expression increased after CFA-induced inflammation and that TDAG8 activation can lead to TRPV1 sensitization, it suggests that high concentrations of protons after inflammation may not only directly activate proton-sensing ion channels (such as TRPV1) to cause pain but also act on proton-sensing GPCRs to regulate the development of hyperalgesia.

## Background

Inflammation induced by tissue injury, infection or tumor growth often accompanies persistent and chronic pain that heightens a pain experience by increasing the sensitivity of nociceptors to both thermal and mechanical stimuli. This phenomenon results, in part, from the production and release of chemical mediators (e.g., protons, adenosine triphosphate, bradykinin, histamine, postaglandin, serotonin) from the primary sensory terminal and from non-neural cells in the environment [1,2]. High local proton concentrations found in inflamed tissues (tissue acidosis) contribute directly to pain and hyperalgesia. The degree of acid-associated pain or discomfort is well associated with the magnitude of acidification, which is attributable to direct excitation or modulation of nociceptive sensory neurons by proton-sensing receptors [3-7].

Several lines of evidence have demonstrated that proton-sensing ion channels are related to acid-associated pain. Mice deficient in the gene transient receptor potential/vanilloid receptor subtype 1 (TRPV1) show reduced sensitivity to thermal stimuli after inflammation [8,9]. Acid-sensing ion channel 3 (ASIC3) is essential for cutaneous and muscle inflammation [10-14]. With peripheral inflammation, the mRNA expression of TRPV1 and ASIC3 is increased in dorsal root ganglia (DRG) and sensitizes their responses [15-20]. Enhanced expression of ASIC3 is probably due to promoter activation by stimulation of inflammatory mediators, especially nerve growth factor (NGF) [18-20]. Although the factors resulting in increased TRPV1 expression and function is unclear, protease-activated receptor 2 was found to sensitize TRPV1 function through protein kinase A (PKA) and protein kinase C (PKC) pathways [21-23]. Interestingly, DRG neurons with increased TRPV1 expression and function after inflammation are non-peptidergic (isolectin B4 [IB_4_]-positive) rather than peptidergic (IB_4_-negative) [17], which suggests that non-peptidergic neurons play important roles in inflammatory pain.

The ovarian cancer G-protein-coupled receptor 1 (OGR1) family, consisting of OGR1, GPR4, TDAG8, and G2A, respond to proton stimulus with full activation at pH 6.4–6.8 [24-27]. These four receptors were found in DRG, and most (75%~82%) are present in small-diameter neurons that are responsible for nociception [28,29]. More than half of these genes are expressed in IB_4_-positive neurons that are involved in inflammatory or neuropathic pain. However, the functions of the OGR1 family subtypes in chronic pain remain unclear.

In this study, we used the inflammatory agents capsaicin, carrageenan, or complete Freund's adjuvant (CFA) to induce peripheral inflammation and examined the change in expression of OGR1 family genes in DRG neurons. OGR1 family subtypes showed differential expression in various types of inflammatory pain (neurogenic, short-term or long-term inflammatory pain). TDAG8 seems to be the major subtype involved in CFA-induced chronic inflammation. Enhanced expression of TDAG8 gene is mainly due to an increase in total number of TDAG8-expressing neurons. Further studies demonstrated that the number of DRG neurons expressing both TDAG8 and TRPV1 genes was increased as well, and TDAG8 activation sensitized TRPV1 response to capsaicin. TDAG8 could be involved in CFA-induced chronic inflammatory pain by regulating TRPV1 function.

## Results

### Differential expression of proton-sensing GPCR genes after induction of peripheral inflammation

To understand whether the proton-sensing OGR1 family is involved in inflammatory pain, mRNA expression was examined in different inflammatory pain states. Peripheral inflammation was generated by intraplantar injection with capsaicin, carrageenan, CFA, or saline. Capsaicin injection induced neurogenic inflammatory pain. Carrageenan and CFA are commonly used models of short-term and long-term chronic inflammatory pain, respectively.

#### Neurogenic inflammatory pain

**Figure 1:**
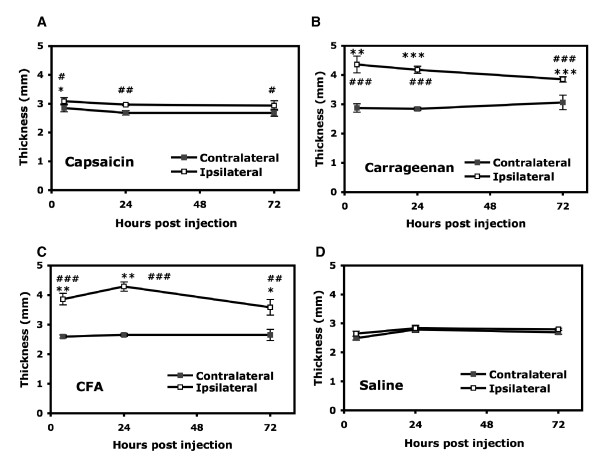
**Thickness of mouse paws after peripheral inflammation**. The wild-type CD1 mice (8–12 week-old) were injected in the right hind paws with 25 μl of capsaicin (100 μg/ml in saline containing 10% ethanol and 0.5% Tween 80, A), carrageenan (20 mg/ml, B), CFA (50% in saline, C), or saline (D). At 4, 24, and 72 hours after injection, the mice were killed and the thickness of injected (ipsilateral) and uninjected (contralateral) paws was measured. All data are presented as mean ± SEM of total tested mice (n = 6–12). Comparison of inflammatory agent-injected and saline-injected animals (*) or between contralateral and ipsilateral paws of agent-injected animals (#) was by *t *test. *# p < 0.05, ** ##p < 0.01, ***###p < 0.001.

**Figure 2:**
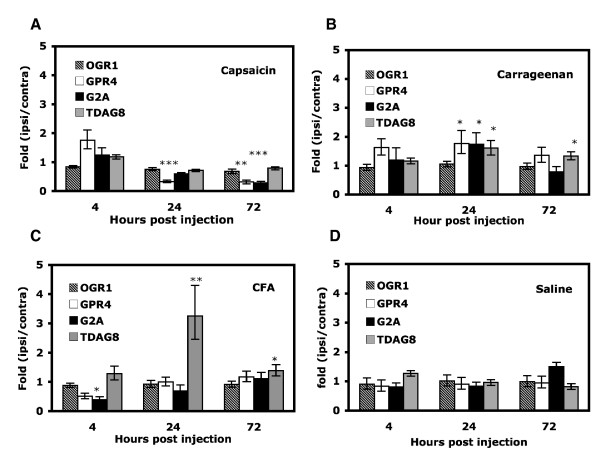
**Gene expression change of OGR1 family genes after peripheral inflammation**. The wild-type CD1 mice (8–12 week-old) were injected at right hind paws with 25 μl of capsaicin (100 μg/ml in saline containing 10% ethanol and 0.5% Tween 80, A), carrageenan (20 mg/ml, B), CFA (50% in saline, C), or saline (D). At 4, 24, and 72 hours after injection, the mice were killed. Lumbar 4–6 DRG ipsilateral and contralateral to injected paws were taken for RNA extraction for quantitative RT-PCR. The contralateral DRG was used as untreated controls. The expression of each gene on the ipsilateral DRG was first normalized to that of mGAPDH and then represented as a relative value to contralateral controls. All data are presented as mean ± SEM of quadruplicates of three experiments (N = 3, n = 3 mice). Comparison between inflammatory agent-injected and saline-injected animals was by *t *test. *p < 0.05, **p < 0.01, ***p < 0.001.

Capsaicin administered locally caused pain-related behaviors. The greatest magnitude of pain-related behaviors was perceived during the first minute after the injection and then rapidly decreased (data not shown). Within 15 minutes, injected mice showed unilateral edema, which gradually decreased. Four hours after injection, the edema lessened, with a paw thickness of 3.09 ± 0.13 mm on the injected (ipsilateral) paw and 2.85 ± 0.13 mm on the uninjected (contralateral) paw (Fig. 1A). A small degree of edema lasted for three days (2.94 ± 0.17 and 2.69 ± 0.12 mm for the ipsilateral and contralateral paws, respectively). Saline-injected controls showed no edema (2.64 ± 0.08 and 2.49 ± 0.06 mm for the ipsilateral and contralateral paws, respectively; Fig. 1D). Surprisingly, after capsaicin injection, GPR4 gene expression decreased 24 hours (2.6~3.4-fold that of the contralateral paw), and such decrease lasted for three days (2.6~4.0-fold at 72 hours). In addition, after capsaicin injection, G2A gene expression decreased (2.7~4.5-fold) at 72 hours after injection. Saline injection produced no increase or decrease in OGR1 family gene expression (Fig. 2D).

#### Short-term inflammatory pain

In carrageenan-induced short-term inflammation, unilateral edema was detected early, within 15 minutes, and peaked at 4 hours after injection (4.36 ± 0.29 and 2.87 ± 0.14 mm for the ipsilateral and contralateral paws, respectively, Fig. 1B). Although decreasing with time, peripheral edema extended to three days (4.18 ± 0.12 mm at 24 hours and 3.85 ± 0.09 mm at 72 hours). At 24 hours after carrageenan injection, GPR4, TDAG8 and G2A expression increased (1.4~2.2-fold for GPR4, 1.4~2.1-fold for G2A, and 1.4~1.9-fold for TDAG8; Fig. 2B). At 72 hours after injection, the expression of GPR4 and G2A returned to baseline, but that of TDAG8 remained at high levels as compared with saline injection.

#### Long-term inflammatory pain

CFA injection induced unilateral peripheral edema 4 hours after injection (3.86 ± 0.19 mm for the ipsilateral paw), smaller than was observed after carrageenan injection (an increase of 46% with CFA injection and 65% with carrageenan injection, as compared with saline injection; Fig. 1A, D). The edema peaked at 24 hours after injection (4.29 ± 0.15 mm), then gradually decreased but remained for at least three weeks (3.33 ± 0.19 mm at 21 days after injection; data not shown). At 24 hours after CFA injection, only TDAG8 expression was increased greatly (2.5~4.3-fold), but the level was reduced at 72 hours (1.2~1.6-fold) (Fig. 2C). In contrast, G2A expression was first decreased at 4 hours after injection (2~3-fold reduction) and gradually returned to basal levels.

**Figure 3:**
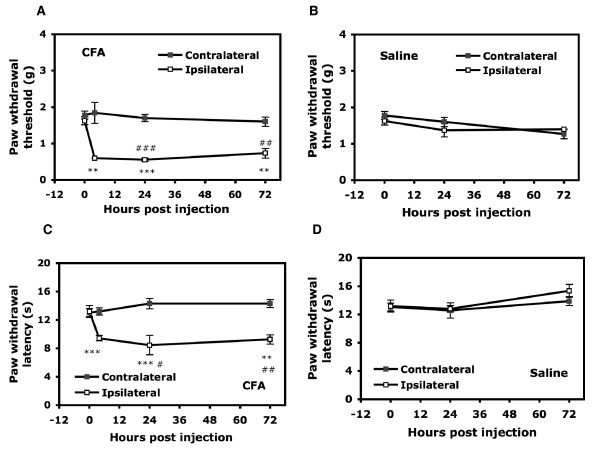
**Mechanical and thermal hyperalgesia in mice after CFA-induced peripheral inflammation**. The wild-type CD1 mice (12 week-old) were injected with 25 μl CFA (50% in saline, A, C), or saline (B, D). The threshold (A, B) and the latency (C, D) of paw withdrawal were measured before injection (t = 0) and after injection (t = 4, 24, and 72 hours). All data are mean ± SEM of total tested mice (n = 6 per group). Comparison between inflammatory agent-injected and saline-injected animals (#) or between contralateral side and ipsilateral side of agent-injected animals (*) was by *t *test. *#p < 0.05, **##p < 0.01, ***###p < 0.001.

Both mechanical and thermal hyperalgesia developed on the ipsilateral paw in CFA-injected mice at 4 hours after injection (Fig. 3). Before injection, the paw withdrawal threshold (50%) for mechanical stimuli (mechanical hyperalgesia) was 1.63 ± 0.11 g on the ipsilateral paw and 1.78 ± 0.11 g on the contralateral paw (0 hour). After CFA injection, the threshold was decreased on the ipsilateral paw to 0.60 ± 0 g at 4 hours and 0.56 ± 0.04 g at 24 hours but remained similar to that in controls on the contralateral paw (1.84 ± 0.29 g at 4 hours and 1.70 ± 0.10 g at 24 hours; Fig. 3A). Mechanical hyperalgesia remained for three weeks (data not shown). Non-noxious mechanical stimulation did not induce hyperalgesia in saline-injected mice (Fig. 3B). The latency of paw withdrawal to heat stimuli (thermal hyperalgesia) was 13.06 ± 0.55 seconds for the contralateral paw and 13.18 ± 0.84 seconds for the ipsilateral paw before injection. At 4 hours after CFA injection, the latency of paw withdrawal to heat was decreased to 9.44 ± 0.38 seconds for the ipsilateral paw and remained at 13.20 ± 0.51 seconds for the contralateral paw (Fig. 3C, D). The decreased latency to heat stimulation lasted for three weeks (data not shown).

### DRG neurons expressing TDAG8 increase in number with CFA-induced inflammation

**Figure 4:**
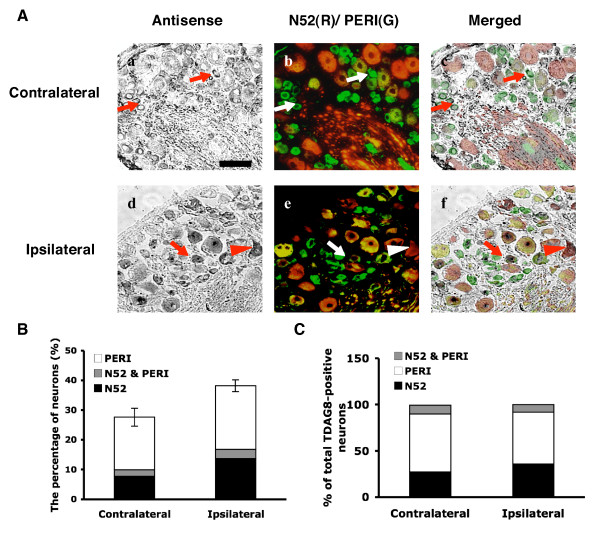
**Number of TDAG8-expressing neurons increases in DRG after CFA-induced inflammation**. After CFA-injection, lumbar 4–5 DRG ipsilateral and contralateral to injected paws were sectioned and hybridized with dig-labeled anti-sense mTDAG8 probes, followed by co-staining with antibodies against peripherin (PERI, green fluorescence) and N52 (red fluorescence). (A) Phase-contrast fields (a, d) are neurons labeled with cRNA probes. Fluorescence images (b, e) show neurons labeled with green (PERI only), red (N52 only), and yellow (PERI and N52). Phase-contrast images and fluorescence images were combined to obtain merged images (c, f). Arrows indicate the peripherin-positive neurons labeled with antisense probes. Arrowheads are the N52-positive neurons labeled with antisense probes. The scale bar is 50 μm. (B) The histogram shows the percentage of total neurons that expressed TDAG8 in PERI, N52, or overlapping subpopulations. (C) The histogram shows the percentage of total TDAG8-positive neurons that co-localized with PERI or N52 markers.

TDAG8 was the only receptor whose expression increased at 24 hours after CFA injection (Fig. 2C). To further determine whether the increased TDAG8 expression was due to an increased number of DRG neurons, we used *in situ *hybridization to examine L4-5 DRG 24 hours after CFA injection. CFA injection did not change the distribution of neurons that were PERI positive (67 ± 3% and 66 ± 2% for the contralateral and ipsilateral DRGs, respectively) and N52 positive (41 ± 3% and 41 ± 2% for the contralateral and ipsilateral DRGs, respectively) (Fig. 4 and Table 1). At 24 hours after CFA injection, 27 ± 2% of total neurons expressed TDAG8 in the contralateral DRG, which was consistent with results for untreated controls found in a previous study [28], whereas the number was increased to 38 ± 2% on the ipsilateral DRG (Fig. 4A, B, Table 1). TDAG8-expressing neurons were increased in number in small- and large-diameter neuron populations. Of PERI-positive neurons, 24 ± 3% expressed TDAG8 on the ipsilateral DRG and 20 ± 2% on the contralateral DRG (Fig. 4A, B). In N52-positive populations, 17 ± 2% showed TDAG8 expression on the ipsilateral DRG and 10 ± 3% on the contralateral DRG (Fig. 4A, B). The distribution of TDAG8-expressing neurons shifted slightly to a population of N52-positive neurons after CFA injection (Fig. 4C), which suggests that TDAG8-expressing neurons were greater in number in medium- to large-diameter neurons.

**Table 1:** Number of TDAG8-expressing neurons in DRG after CFA-induced peripheral inflammation

	**IR Neurons/total neurons**	**TDAG8-labeled neurons/total neurons**	**TDAG8-labeled neurons/total TDAG8-labeled neurons**
**Contralateral DRG**			
Total		27 (25~30)	
N52	41 (37~45)	10 (7~12)	36 (30~43)
PERI	67 (64~70)	20 (17~21)	72 (67~78)
N52 & PERI	8 (5~11)	2 (1~3)	9 (6~13)

**Ipsilateral DRG**			
Total		38 (36~40)	
N52	41 (39~43)	17 (15~19)	44 (41~48)
PERI	66 (65~68)	24 (23~27)	64 (61~68)
N52 & PERI	8 (6~10)	3 (2~4)	8 (6~13)

Percentage of labeled neurons in total DRG neuron populations with 95% confidence intervals. The number of total cells counted was 900~1500. PERI = peripherin. IR = immunreactive.

**Figure 5:**
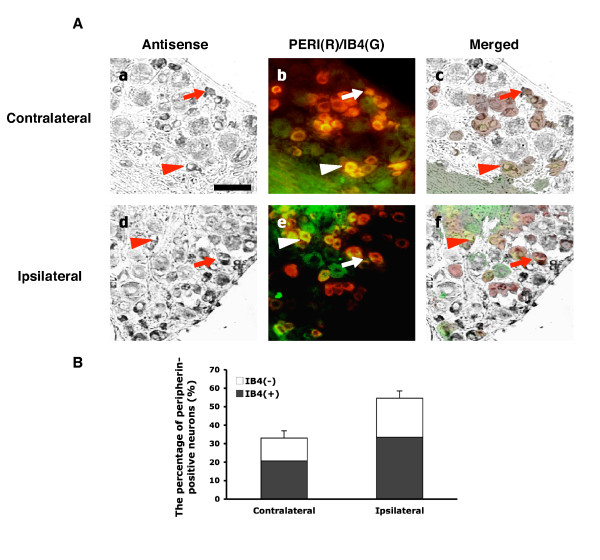
**Number of TDAG8-expressing neurons increases in both IB_4_-positive and -negative neurons after CFA-induced inflammation**. After CFA-injection, lumbar 4–5 DRG ipsilateral and contralateral to injected paws were sectioned and hybridized with dig-labeled anti-sense mTDAG8 probes, followed by staining with antibodies against peripherin (PERI, red fluorescence) and IB_4_-FITC conjugates (IB_4_, green fluorescence). (A) Phase-contrast fields (a, d) are neurons labeled with cRNA probes. Fluorescence images (b, e) show neurons labeled with red (PERI only), green (IB_4 _only), and yellow (PERI and IB_4_). Phase-contrast images and fluorescence images were combined to obtain merged images (c, f). Arrows indicate the peripherin-positive neurons labeled with antisense probes. Arrowheads are the PERI and IB_4 _co-expressing neurons labeled with antisense probes. The scale bar is 50 μm. (B) The histogram shows the percentage of total PERI neurons that expressed TDAG8 in IB_4_-positive or -negative subpopulations.

TDAG8 expression was further examined in peptidergic (IB_4_-negative) and non-peptidergic (IB_4_-positive) sub-populations. CFA-induced inflammation did not alter the distribution of neurons in nociceptors that were IB_4 _positive (52 ± 4% on the contralateral DRG and 56 ± 5% on the ipsilateral DRG) or negative (48 ± 4% and 44 ± 5% on contralateral and ipsilateral DRG, respectively) (Fig. 5A, B and Table 2). Of the neurons labeled with PERI, 33 ± 4% of IB_4_-positive neurons expressed TDAG8 on the ipsilateral DRG as compared with 21 ± 4% on the contralateral DRG at 24 hours after CFA injection. TDAG8-expressing neurons were also increased in number in the IB_4_-negative population (12 ± 3% and 21 ± 4% for the contralateral and ipsilateral DRGs, respectively) (Fig. 5B, Table 2). Accordingly, TDAG8-expressing neurons were increased in number in both IB_4_-positive and -negative populations after CFA injection.

**Table 2:** Number of TDAG8-expressing neurons in IB4-positive and -negative neurons after CFA-induced inflammation

	**IR neurons/total PERI-IR neurons**	**TDAG8-labeled neurons/total neurons**	**TDAG8 probe-labeled neurons/total TDAG8-labeled neurons**
**Contralateral DRG**			
PERI (+)		33 (28~37)	59 (54~64)
IB_4 _(+)		41 (31~43)	48 (44~52)
PERI (+) & IB_4 _(+)	52 (48~56)	21 (17~25)	63 (59~68)
PERI (+) & IB_4 _(-)	48 (44~52)	12 (9~15)	37 (33~42)

**Ipsilateral DRG**			
PERI (+)		48 (44~52)	61 (56~66)
IB_4 _(+)		57 (52~62)	49 (43~52)
PERI (+) & IB_4 _(+)	56 (51~61)	33 (28~37)	69 (65~74)
PERI (+) & IB_4 _(-)	44 (39~49)	21 (17~25)	43 (38~47)

Percentage of labeled neurons in total DRG neuron populations with 95% confidence intervals. The number of total cells counted was 900~1000. PERI = peripherin. IR = immunoreactive.

### TDAG8 activation increases levels of intracellular cAMP

**Figure 6:**
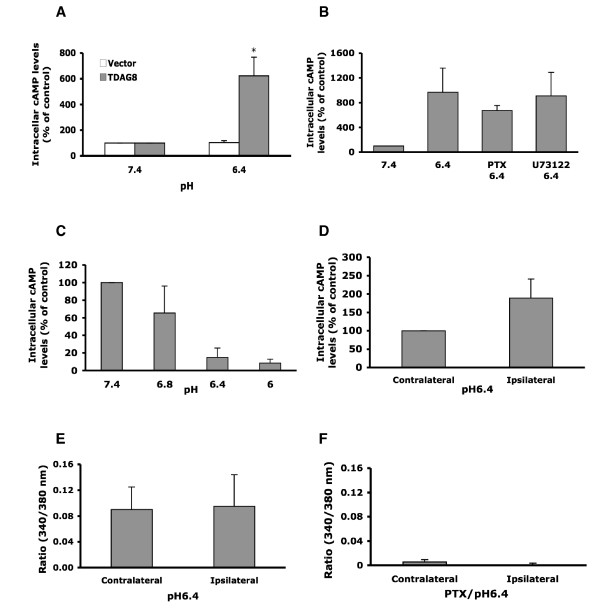
**Accumulation of cAMP by cells exposed to acidic pH**. (A) HEK293T cells were transfected with pIRES-GFP-TDAG8 or pIRES-GFP plasmids for 36 hours. Transfected cells were exposed to pH7.4 and 6.4 buffers for 30 minutes at 37°C in the presence of RO201724 for detection of intracellular cAMP. All values were normalized with pH 7.4 vector control (as 100%). Data are obtained from at least three independent experiments performed in duplicate. Comparison between vector- and TDAG8-transfected cells was by *t *test, *p < 0.05. (B) The accumulation of cAMP in TDAG8-transfected HEK293T cells pre-treated with PTX for 4 hours or U73122 for 15 minutes at 37°C before exposure to pH 6.4 buffer for 30 minutes. All values were normalized with pH 7.4 control (as 100%). (C) The pH-dependent cAMP accumulation in DRG primary cultures. Primary DRG cultures were exposed to pH7.4, 6.8, 6.4 and 6.0 buffers for 30 minutes at 37°C in the presence of RO201724 for detection of intracellular cAMP. The cAMP levels at pH 7.4 were a control (= 100%). (D) The cAMP levels in DRG cultures from CFA-injected mice after exposure to pH 6.4 buffer for 30 minutes at 37°C in the presence of RO201724. The cAMP levels from contralateral DRG were a control (100%). (E) Intracellular [Ca^2+^] change in DRG cultures from CFA-injected mice after transient exposure to pH 6.4 buffer. Peak values of [Ca^2+^] response (approximately 20 seconds after the addition of agonists) are presented as histograms. All data are presented as mean ± SEM (n = 12 cells). (F) Intracellular [Ca^2+^] change in DRG cultures from CFA-injected mice. DRG cultures were transiently exposed to pH 6.4 buffer after pre-treatment with PTX for 2 hours. Peak values of [Ca^2+^] response are presented as histograms. All data are presented as mean ± SEM (n = 7–12 cells).

Whether increased TDAG8 expression enhances TDAG8 function after inflammation has remained unclear. We first examined proton signaling in HEK293T cells transfected with TDAG8. Consistent with previous studies [26], TDAG8-expressing cells responded to protons and induced increased levels of intracellular cAMP. The cAMP response peaked at pH 6.6~6.4 (data not shown). Levels of cAMP with pH 6.4 was 6-fold higher that with pH 7.4 (Fig. 6A). To confirm whether the signaling elicited by TDAG8 is through the Gs protein, we used two inhibitors: pertussis toxin (PTX) blocks Gi protein-mediated signaling, and U73122 inhibits phospholipase Cβ (PLCβ), which is activated by Gq or Gi protein. Treatment with PTX or U73122 did not inhibit the cAMP signaling elicited by TDAG8, which suggests that cAMP accumulation is through activation of Gs protein (Fig. 6B).

The accumulation of intracellular cAMP was further examined in primary DRG cultures. Surprisingly, cAMP accumulation declined with acid stimulation in untreated DRG cultures (Fig. 6C), which suggests a Gi-mediated signaling elicited by acid stimulation. At 24 hours after CFA injection, DRG cultures from ipsilateral paws showed higher levels of cAMP after pH 6.4 stimulation (~2-fold increase) than those from contralateral paws (Fig. 6D), although the increase was not great. Since acid-induced Gi-signaling was found in primary culture, increased cAMP levels could be resulted from enhancement of Gs-signaling or reduction of Gi-signaling. To clarify this point, intracellular [Ca^2+^] ([Ca^2+^]_i_) was examined. After pH6.4 stimulation, the same levels of [Ca^2+^]_i _were found in contralateral and ipsilateral DRG cultures from CFA-injected mice (Fig. 6E), suggesting that CFA-injection did not enhance or reduce acid-induced Gi- or Gq-signaling. PTX treatment before acid stimulation inhibited [Ca^2+^]_i _levels in both ipsilateral and contralateral DRG cultures (Fig. 6F), indicating that [Ca^2+^]_i _increase found in contralateral and ipsilateral DRG cultures was due to Gi-signaling. Accordingly, increased cAMP levels observed in ipsilateral DRG cultures were primarily due to enhancement of Gs-signaling.

### Co-localization of TDAG8 with TRPV1 increased after inflammation

**Figure 7:**
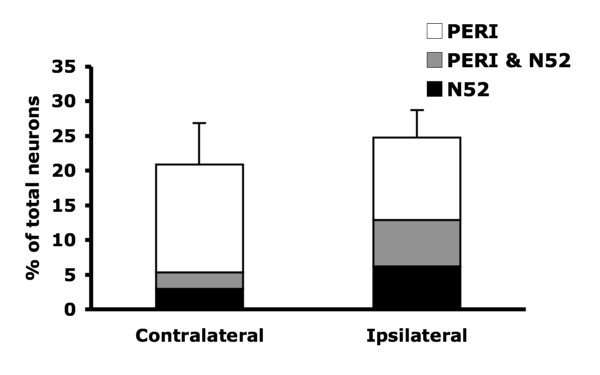
**Number of DRG neurons co-expressing TDAG8 and TRPV1 after CFA-induced inflammation**. DRG tissues were contiguously sectioned at 6 μm and hybridized with dig-labeled antisense cRNA probes, then co-stained with antibodies against PERI and N52. Each pair of sections was hybridized with two different gene cRNA probes. The histogram shows the percentage of total DRG neurons that expressed both TDAG8 and TRPV1 genes.

Although TDAG8 mRNA expression was increased after CFA-induced peripheral inflammation, the function of TDAG8 in inflammatory pain is unclear. TDAG8 likely regulates other molecules (such as TRPV1 or ASICs) to influence mechanical or thermal hyperalgesia [8-14]. Given that 40% of TRPV1-positive neurons express TDAG8 [28,29], we wondered whether co-localization of TRPV1 and TDAG8 is changed after CFA injection. Co-localization of TDAG8 and TRPV1 was examined in pairs of continuous 6-μm DRG sections. Each pair was hybridized with antisense cRNA of TDAG8 and TRPV1, then co-stained with antibodies against N52 and PERI. In the total DRG population, 40 ± 5% neurons expressed TDAG8, 41 ± 5% expressed TRPV1, and 21 ± 4% expressed both (Table 3). At 24 hours after CFA injection, 51 ± 7% of total neurons expressed TDAG8, 47 ± 7% expressed TRPV1, and 25 ± 6% expressed both. After inflammation, an increased number of PERI- and N52-positive neurons began to express TDAG8 (Table 3), but mainly the N52-positive population showed an increase in number of neurons expressing TRPV1 (from 12 ± 3% to 23 ± 6%, Table 3). CFA injection did not produce a significant increase in total number of neurons expressing both genes (from 21 ± 4% to 25 ± 6%), but medium- to large-diameter neurons showed a significant increase (8%) (Fig. 7, Table 3). Surprisingly, the number of small-diameter neurons (PERI-positive only) that expressed both TDAG8 and TRPV1 genes was slightly decreased (4%) after injection.

**Table 3:** Number of DRG neurons co-expressing TDAG8 and TRPV1 after CFA-induced inflammation

	**TDAG8-labeled neurons/total neurons**	**TRPV1-labeled neurons/total neurons**	**TRPV1 & TDAG8-labeled neurons/total neurons**
**Contralateral DRG**			
Total	40 (35~45)	41 (36~46)	21 (16~24)
PERI	35 (30~39)	32 (28~36)	18 (14~21)
N52	8 (5~11)	12 (9~15)	5 (3~7)
N52 & PERI	3 (2~5)	4 (3~6)	2 (1~4)

**Ipsilateral DRG**			
Total	51 (44~58)	47 (40~54)	25 (19~32)
PERI	39 (33~46)	33 (26~40)	19 (13~24)
N52	21 (16~27)	23 (17~29)	13 (9~18)
N52 & PERI	9 (5~14)	9 (5~14)	7 (4~11)

Percentage of neurons that expressed single gene or two genes in total DRG neuron populations with 95% confidence intervals. The number of total cells counted was 150~350. PERI = peripherin

### TDAG8 activation sensitizes TRPV1 response to capsaicin

**Figure 8:**
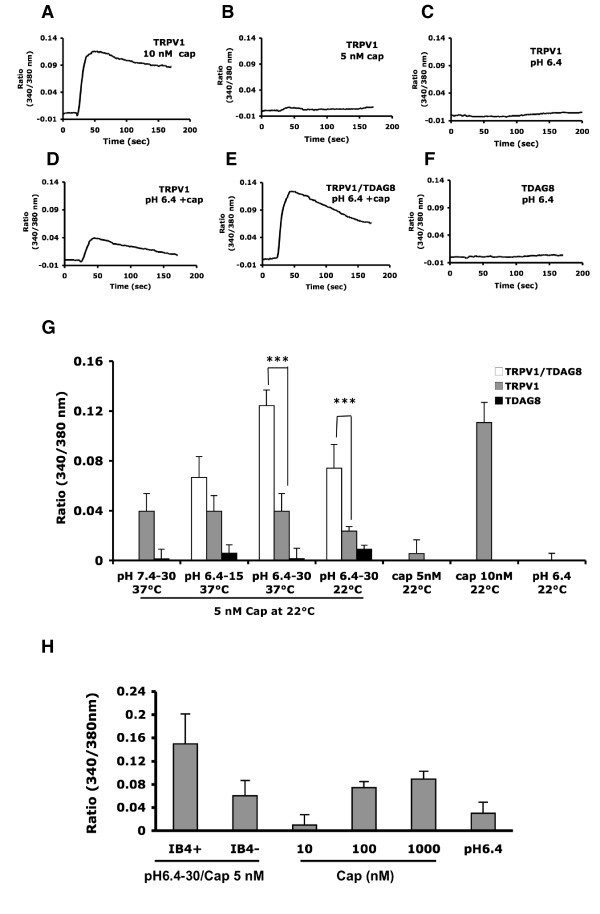
**TDAG8 activation sensitizes TRPV1 response to capsaicin**. (A-G) HEK293T cells were transfected with TDAG8-pIRES-GFP, TRPV1-pIRES-GFP, or both plasmids. After 36 hours, cells were exposed to pH 7.4, 6.4 or vehicle for 15 or 30 minutes at 37°C or at 22°C and then washed with pH7.4 buffer, followed by addition of 5 nM of capsaicin at room temperature. Time courses of [Ca^2+^]_i _signals following addition of different agonists (A-F). Intracellular [Ca^2+^] change in HEK293T cells (G). Peak values of [Ca^2+^]_i _response (approximately 20 seconds after the addition of agonists) are presented as histograms. All data are presented as mean ± SEM (n = 14–23 cells). Comparison between TRPV1 and TRPV1/TDAG8-transfected cells was by *t *test, ***p < 0.005. (H) Intracellular [Ca^2+^] change in DRG cultures. Primary DRG cultures were transiently exposed to different concentrations of capsaicin or pH6.4 at room temperature. Some cultures were pre-treated with pH6.4 buffer for 30 minutes at 37°C and then washed with pH7.4 buffer, followed by addition of 5 nM of capsaicin at room temperature. Peak values of [Ca^2+^]_i _response (approximately 20 seconds after the addition of agonists) are presented as histograms. All data are presented as mean ± SEM (n = 12–18 cells).

We further tested whether TDAG8 activation sensitizes the TRPV1 response to capsaicin. The addition of 10 nM capsaicin to the cells produced a rapid increase in [Ca^2+^]_i _levels (0.110 ± 0.016) in TRPV1-expressing cells, but the addition of 5 nM capsaicin (0.001 ± 0.011) or pH 6.4 buffer (0.000 ± 0.006) did not induce a significant response (Fig. 8A, B, C, G). Pre-treatment with pH 6.4 buffer for 30 minutes before the addition of 5 nM capsaicin (pH 6.4–30/cap) slightly increased [Ca^2+^]_i _in TRPV1-expressing cells (0.039 ± 0.014), as compared with adding capsaicin only (Fig. 8B, D, G). A similar magnitude of increase was also found with pH 7.4–30/cap and pH 6.4–15/cap treatments (Fig. 8G). The same treatment (pH 6.4–30/cap) applied in cells co-expressing both TDAG8 and TRPV1 induced a 3-fold enhanced [Ca^2+^]_i _response (0.124 ± 0.013), as compared with [Ca^2+^]_i _signaling induced by TRPV1-expressing cells (Fig. 8E, G). TDAG8-expressing cells showed no change in [Ca^2+^]_i _levels after exposure to pH 6.4 or 7.4 buffers (Fig. 8F, G). Pre-treatment with pH6.4 or 7.4 buffers for 30 minutes had a slight [Ca^2+^]_i _increase in TRPV1-expressing cells. It could be resulted from the temperature effect as pre-treatment was done at 37°C. To avoid temperature influence, acid pre-treatment was performed at 22°C. Significant [Ca^2+^]_i _increase (0.074 ± 0.019; 3-fold increase) was observed in TRPV1 and TDAG8 co-expressing cells, as compared with [Ca^2+^]_i _response (0.024 ± 0.004) seen in TRPV1-expressing cells (Fig. 8G). The similar experiments were done in primary DRG culture. As showed in Fig. 8H, acid pre-treatment (pH6.4) for 30 minutes before the addition of 5 nM capsaicin increased [Ca^2+^]_i _levels in both IB_4_-positive and negative DRG neurons. IB_4_-positive neurons showed a higher [Ca^2+^]_i _increase.

## Discussion

OGR1 family members were recently found in DRG, and most (75%~82%) are in small-diameter neurons that are responsible for nociception [28,29]. Here, we demonstrated that OGR1 family subtypes are expressed differently in different inflammatory pain states, which suggests that they are involved in neurogenic, short-term and long-term chronic inflammatory pain. The receptor TDAG8 with particularly increased expression after CFA-induced inflammation. Increased mRNA levels were due to the increased number of neurons expressing TDAG8. Given that the number of neurons expressing both TDAG8 and TRPV1 genes was increased as well and that TDAG8 activation sensitized TRPV1 responses to capsaicin, TDAG8 is likely involved in long-term chronic inflammatory pain by sensitizing TRPV1 function.

Capsaicin, carrageenan, or CFA administered locally causes pain-related behaviors, which is accompanied by erythema and edema. Consistent with previous studies [30], capsaicin injection induces neurogenic inflammation. Significant edema developed rapidly within 5 minutes after the administration of capsaicin but was reduced gradually, which indicates an acute inflammatory response. Both carrageenan and CFA injection induced chronic inflammation with higher magnitude and longer duration of edema than did capsaicin injection. Although carrageenan induced edema with a magnitude similar to that produced by CFA, the edema produced by carrageenan peaked in level earlier at 4 hours and remained for a week. The edema induced by CFA injection was fully developed at 24 hours but lasted longer, for 3 weeks. This finding suggests that CFA injection induces long-term chronic inflammation with a peak at 24 hours, whereas carrageenan causes short-term chronic inflammation with a peak at 4 hours.

Hyperalgesia development seems to be well associated with edema development. Previous studies of humans, rats or mice have demonstrated that hyperalgesia induced by capsaicin injection is dose dependent and develops rapidly within 15 minutes after injection [31-35]. Our observations are consistent with these studies: low-dose capsaicin (2.5 μg/paw) administered locally induced pain-related behaviors within 5 minutes; heat hyperalgesia disappeared in 15 minutes after injection, but mechanical hyperalgesia lasted for hours. Carrageenan induced a sub-chronic inflammatory pain. The peak of hyperalgesia was at 4 hours, and hyperalgesia was extended to several days, which is consistent with previous studies [36-38]. In the CFA-injection model, both thermal and mechanical hyperalgesia developed fully within 24 hours after injection and extended to three weeks, which is similar to previous observations [37,38].

The OGR1 family were expressed differently in these three inflammatory pain models. After capsaicin injection, GPR4 gene expression was reduced to 2.5~3-fold the basal levels at 24 hours and remained so for three days, whereas G2A transcripts were significantly reduced 72 hours after injection. Capsaicin-induced edema and hyperalgesia developed within 15 minutes. Down-regulation of GPR4 and G2A is unlikely to have a direct influence on the formation of hyperalgesia or edema. Possibly, reduced levels of GPR4 and G2A mRNA prevent the extension of edema and hyperalgesia. Administration of high-dose capsaicin to adult rats or the application of capsaicin to primary sensory neurons induces a subpopulation of DRG neuron death [39-41]. Although capsaicin dosage used in our studies was lower than that used in rats, it is possible that such dosage may have some influence on neurons that expressed GPR4 and G2A genes. GPR4-expressing neurons could be more sensitive to capsaicin administration than neurons expressing G2A. The *in situ *hybridization experiments had confirmed that the number of GPR4-positve neurons was decreased (9% of decrease) after capsaicin injection (data not shown). Capsaicin-injection also induced a slight reduction in the number of PERI-positive neurons. Alternatively, down-regulation of GPR4 and G2A genes is due to functional desensitization.

GPR4, G2A, and TDAG8 transcripts were increased in levels 24 hours after carrageenan injection, but only TDAG8 expression was increased after CFA injection. In both models, the transcripts of the three genes increased in levels at 24 hours when edema and hyperalgesia were already developed. GPR4, G2A, and TDAG8 seem to maintain edema and hyperalgesia rather than induce edema and hyperalgesia. TDAG8 was identified from apoptotic immature thymocytes, where its expression is up-regulated, which suggests that TDAG8 is involved in immune cell development [42]. Although TDAG8 expression is required for the production of cAMP in immune cells, mice lacking TDAG8 have normal immune cell development [43,44]. In endothelial cells, G2A expression blocks NF-kB activation and chemokine expression, thus inhibiting macrophage accumulation [45], which suggests that G2A expression may have a protective role for prevention of early events of inflammation. GPR4 is present in the endothelial cells of blood vessels, and mice lacking GPR4 show vascular abnormalities, which suggests that GPR4 has a role in vascular growth and vascular stability [46]. Vascular stability is important for leukocyte adhesion and function [47]. TDAG8, G2A, and GPR4, were all previously suggested to have pro-inflammatory or anti-inflammatory roles. Whether they have similar roles in nociceptors is unclear. OGR1 is the only receptor whose expression did not change in any inflammatory pain models we tested. Since the level of OGR1 in DRG is the highest among the family members [28], OGR1 is likely the pH sensor for physiological conditions, whereas other family members are responsible for different pathological conditions. However, OGR1 protein and function could still be enhanced after inflammation despite no change in mRNA expression.

TDAG8 is the major proton-sensing GPCR showing increased expression after CFA-induced inflammation. Enhanced mRNA levels were due to an increase in the total number of neurons expressing TDAG8. A considerable number of A-fiber neurons began to express TDAG8 after CFA injection. We also found that the number of TRPV1-expressing neurons was increased in medium- and large-diameter neurons after inflammation, consistent with previous results in rats [16]. The total number of A-fiber neurons that expressed both TDAG8 and TRPV1 was largely increased after CFA injection. The peripheral large-diameter neurons are known to respond to innocuous mechanical stimuli, whereas medium-diameter (A-delta fiber) neurons are classified into two types, both of which respond to intense mechanical stimuli but have differential responsiveness to heat [1,48]. The plasticity of A-fiber neurons is an important mechanism that causes hyperalgesia following inflammation, therefore, increased TDAG8 expression in A-fiber could be involved in mechanical and thermal hyperalgesia by regulating TRPV1 function.

In small-diameter neurons, TDAG8-expressing neurons were increased in number in both IB_4_-positive and -negative nociceptors. IB_4_-positive and -negative nociceptors have not only biochemical and anatomical differences but also distinct functions owing to their physiological properties [49,50]. IB_4_-negative nociceptors are primarily responsible for the initial nociceptive response to proton, capsaicin and noxious heat; whereas the responsiveness of IB_4_-positive neurons to capsaicin is increased after proton stimulation [51]. Breese et al. [17] proposed that peripheral inflammation sensitizes the responses of IB_4_-positive neurons to protons and capsaicin, which is due to enhanced expression and function of TRPV1. The function of IB_4_-positive neurons in inflammation has recently been of interest. Increased TDAG8 expression in IB_4_-positive neurons after peripheral inflammation may increase the sensitivity of IB_4_-positive neurons to proton and capsaicin, through modulating the functions of ASICs or TRPV1.

Cells co-expressing TDAG8 and TRPV1 showed significantly enhanced responsiveness of cells to 5 nM of capsaicin after pre-treatment with proton (pH 6.4), as compared with cells expressing TRPV1 only. This finding suggests that proton potentiates TRPV1-mediated [Ca^2+^]_i _increase in the presence of TDAG8. The large increase of [Ca^2+^]_i _is unlikely directly induced by TDAG8 because cells expressing TDAG8 alone did not show elevated [Ca^2+^]_i _levels but, rather, accumulated intracellular cAMP levels. The enhanced response of TRPV1 to capsaicin was possibly due to proton-induced TDAG8 activation. Similar results were found in primary culture. Acid pre-treatment (pH 6.4) increased the responsiveness of DRG neurons to capsaicin. IB_4_-positive neurons had a higher sensitivity than IB_4_-negative neurons. Given that IB_4_-positive neurons had more TDAG8 expression than IB_4_-negative neurons after CFA-injection, the enhanced sensitivity of IB_4_-positive neurons to capsaicin may be due to increased TDAG8 expression.

Interestingly, 5 nM capsaicin induced a slight increase in [Ca^2+^]_i _levels in TRPV1-expressing cells after pre-treatment with different pH buffers (pH 7.6 and 6.4) at 37°C for 30 minutes. Such increase is likely due to the temperature effect, because application of different pH buffers or different times showed similar results. Pre-treatment at 22°C reduced such [Ca^2+^]_i _increase. The temperature influence on the TRPV1 response to capsaicin was previously demonstrated [52].

CFA-induced inflammation selectively increases the responsiveness of IB_4_-positive neurons to protons and capsaicin because of the enhanced response of TRPV1 [17]. Our observation could provide an explanation for the enhanced TRPV1 function in IB_4_-positive neurons after inflammation. In IB_4_-positive neurons, TDAG8 responds to extracellular protons leads to cAMP accumulation to activate PKA directly. PKA modulates TRPV1 function by phosphorylation [53,54]. Alternatively, TDAG8-activation may act on other second messenger kinases, such as PKCε through the cAMP-Epac-PKCε pathway. The cAMP-Epac-PKCε pathway is important to IB_4_-positive neurons to mediate inflammatory pain [55]. PKCε not only modulates TRPV1 function but also regulates prolonged response of the chronic inflammatory pain [54,56-58]. Therefore, TDAG8 could be involved in sensitization of TRPV1 function in IB_4_-positive neurons and in the development of the chronic inflammatory pain state through the cAMP-PKCε signaling. Accordingly, peripheral inflammation induces a local increase of protons, leading to TDAG8 activation. TDAG8 activation elevates cAMP levels, activating PKA and PKCε. PKA or PKCε sensitizes TRPV1 or other ion channels to cause hyperalgesia.

Although the number of neurons expressing TDAG8 increased after inflammation, the cAMP accumulation by TDAG8 was not enhanced significantly. We used cAMP assays here was to measure total cAMP levels in a culture population. Given that we found only an 11% increase in TDAG8-expressing neurons after inflammation and that DRG neurons are heterogeneous, our finding of no large increase in cAMP levels in DRG cultures after CFA injection is not surprising. Interestingly, cAMP levels were lower in primary culture after stimulation with pH 6.4 than with pH 7.4. This decrease is pH dependent and raises a possibility that increased cAMP levels observed after CFA injection is due to a reduction of Gi-signaling rather than an enhancement of Gs-signaling. Given that acid-induced [Ca^2+^]_i _increase did not change in contralateral or ipsilateral DRG neurons after CFA-injection and such increase was from Gi-signaling, increased cAMP levels after inflammation are more likely due to enhanced Gs-signaling.

Previous studies have reported that proton-sensing GPCRs mediate Gs and Gq signaling pathways [24-27]. Unexpectedly, we observed pH-dependent Gi responses in DRG culture. Later studies in different tissues or cells have found that OGR1 and GPR4 can mediate more than one type of G-protein signaling [59-61]. The OGR1 family subtypes may also mediate Gi signaling. Alternatively, OGR1 family members forming heterodimers for function provides an explanation for why pH-dependent Gi signaling is present in DRG primary culture. About 31%~40% nociceptors express at least two OGR1 family genes [28]. Such a high degree of co-localization may reflect that a heterodimer formation between OGR1 family members is necessary for their function, because a dimerization of GPCRs is now accepted as a functional unit for ligands [62,63]. A GPCR heterodimer might have signaling pathways different from those of a homodimer [62]. A heterodimeric receptor likely switches Gs or Gq signaling to Gi signaling.

## Conclusion

This is the first study to systematically explore the expression changes of proton-sensing GPCRs (the OGR1 family) at different times and in different inflammatory pain models (capsaicin, carrageenan, or CFA). Each subtype of the OGR1 family was expressed differently, which may reflect differences between models in duration and magnitude of hyperalgesia. This finding also implies the complexity of the mechanism of inflammatory pain. As demonstrated here, TDAG8 activation can lead to TRPV1 sensitization, and TDAG8 expression increased after CFA-induced inflammation. Our results suggest that high concentrations of protons after inflammation may not only directly activate proton-sensing ion channels (such as ASIC3 and TRPV1) to cause pain but also act on proton-sensing GPCRs to regulate the development of hyperalgesia or to enhance the sensitivity of neurons.

## Methods

### Inflammation experiments and tissue collection

Male CD-1 mice (8–12 weeks old) (were bred in animal house in the National Central University, Taiwan) underwent intraplantar injection with 25 μl of saline, CFA (50% in saline), carrageenan (20 mg/ml in saline), or capsaicin (100 μg/ml in saline containing 10% ethanol and 0.5% Tween 80). At 4, 24, and 72 hours after injection, the mice were killed and paw thickness was measured. Lumbar 4–6 DRG ipsilateral and contralateral to injected paws were removed for RNA extraction, with the ganglia from uninjected paws serving as negative controls. L4-5 DRG were frozen for cryosectioning. The animal experimental procedures were approved by the Animal Care and Use Committee at the National Central University, Taiwan.

### Behavioral tests

To assess mechanical nociceptive responses, animals were tested for withdrawal thresholds to mechanical stimuli (von Frey filaments, Touch-Test, North Coast Medical, Inc., Morgan Hill, CA) applied to the plantar aspect of the hindpaw. Mice (n = 6 per group) were placed on a wire mesh platform in transparent plexiglas chambers (10 × 8 × 10 cm/chamber), allowed to habituate for 2 hours each day and trained for 3 days before the test. At 4, 24, and 72 hours after mice were injected with inflammatory agents or saline, we applied a series of von Frey fibers (0.4, 0.6, 1.0, 1.4, 2.0 g), in ascending order beginning with the finest fiber, through the wire mesh onto the plantar surface of both hindpaws of mice. A withdrawal response was considered valid only if the hindpaw was removed completely from the platform. If the paw withdrawal response was ambiguous, the application of fibers was repeated. For each paw, a von Frey fiber was applied 5 times at 5-second intervals. The threshold was when paw withdrawal was observed in more than 3 of 5 applications.

Animals were also tested for thermal nociceptive response to radiant heat applied to the plantar surface of the paw [36]. Mice (n = 6 per group) were allowed to habituate for at least 2 hours in transparent plexiglass chambers (10 × 8 × 10 cm/chamber) on a glass floor before testing. At 4, 24, and 72 hours after mice were injected with inflammatory agents or saline, we stimulated the plantar surface of mouse hindpaws with a light bulb (40% intensity, 305 mW/cm^2^). The latency to withdrawal of the paw from radiant heat was measured. Measurements from three trials at 1-minute intervals in each paw were averaged. We obtained mean basal withdrawal latencies of 12~15 seconds in uninjected mice.

### RNA preparation and quantitative RT-PCR

DRG RNA extraction involved use of the RNeasy kit (Qiagen, Valencia, CA), according to the manufacturer's instructions. RNA was reverse transcribed by use of Superscript II RT (Invitrogen, Carslbad, CA) with oligo dT (Invitrogen). Derived cDNA was used as a template for quantitative RT-PCR.

The reaction mixture (25 μl) included 6 μl of 2× master mix (containing SYBR green I and AmpliTaq Gold DNA polymerase [Applied Biosystems, Foster City, CA]), 100 nM each of primers and cDNA. The primer sets for each gene were as follows: OGR1 (151 bp), 5'-gacgataccagcccaagtgt-3' (forward) and 5'-gctgttatccctagccacca-3' (reverse); GPR4 (199 bp), 5'-cttcctcagcttcccaagtg-3' (forward) and 5'-cctgggcctcctttctaaac-3' (reverse); G2A (166 bp), 5'-aagtgtccagaatccacacagggt-3' (forward) and 5'-agtaaacctagcttcgctggctgt-3' (reverse); and TDAG8 (197 bp), 5'-atagtcagcgtcccagccaac-3' (forward) and 5'-cgcttcctttgcacaaggtg-3' (reverse). The internal control was also measured from the same samples [mGAPDH, NM_001001303, 233 bp, primers: 5'-ggagccaaacgggtcatcatctc-3' (forward) and 5'-gaggggccatccacagtcttct-3' (reverse)].

For each assay, three independent preparations were run in quadruplicate. The DRG pool had at least 9 ganglia. The thermal cycling conditions were 95°C for 10 min, followed by 40 cycles of 95°C for 15 s, and 60°C for 1 min. PCR reactions and product detection were carried out in the ABI Prism 7300. The amplified product was detected by measurement of SYBR green I, which was added to the initial experiment mixtures. The threshold cycle (Ct) values obtained from the experiments indicated the fractional cycle numbers at which the amount of amplified target reached a fixed threshold. The Ct values of both the targets and internal reference (mGAPDH) were measured from the same samples, and the expression of the target genes relative to that of mGAPDH was calculated by the comparative Ct method.

### *In situ *hybridization and immunohistochemistry

Excised lumbar DRG tissue was immediately put into freezing solution. Serial sections 12 μm thick were cut by use of a cryostat (Leica microsystem 3510S, Bensheim, Germany). For colocalization experiments, several pairs of contiguous sections 6 μm thick were prepared. Sections were attached to slides coated with 3-aminopropyltrithozysilane (2%). After fixation with 4% paraformaldehyde at 4°C for 30 min, sections were acetylated for 10 min with 0.12% (v/v) triethanolamide and 0.25% (v/v) acetic anhydrides (all Merck). After pre-incubation with hybridization buffer (50% formamide, 4× SSC, 2× Denhardt's solution, and 50 μg/ml tRNA) for 2 h at room temperature, the digoxigenin-UTP (dig, Roche)-labeled complementary RNA (cRNA) probes diluted in hybridization buffer were denatured and hybridized to the DRG sections overnight at 65°C. The dig-labeled probes were generated by *in vitro *transcription with T7 and T3 polymerases (Roche) from nucleotide sequences as follows: nucleotides 1183~1599 (417 bp) for TDAG8 (NM_008152), and nucleotides 2519~2651 (132 bp) for TRPV1 (ENSMUSG00000005952). Following the hybridization, the slides underwent high-stringency washing cycles: four times of 2× SSC (20× SSC stock: 3 M NaCl and 0.3 M sodium citrate, pH 7.0) at 72°C for 10 min, three times of pre-warmed 2× SSC at 72°C for 30 min, three times of pre-warmed 0.1× SSC at 72°C for 60 min, and twice of 0.2× SSC at room temperature for 10 min. After the washing, the dig-labeled cRNA probes were detected with use of an alkaline phosphatase-conjugated anti-dig antibody (Roche) by incubation for 1 h at room temperature. Development of signals involved use of a mix of nitro-blue tetrazolium chloride, 0.45%, and 5-bromo-4-chloro-3'-indolyphosphate p-toluidine salt, 0.35% (Sigma, St. Louis. MO). The specificity of hybridization signals was confirmed by a control study involving sense cRNA probes for each gene.

After detection of hybridization signals, sections were washed with 1× PBS and then co-stained with various combinations of primary antibodies, followed by suitable secondary antibodies. All antibodies were diluted in 1× PBS containing 1% BSA. All antibody incubations were carried out at 4°C overnight. Primary antibodies were against N52 (1:500, Sigma) or peripherin (PERI; 1:500, Chemicon). Secondary antibodies were goat-anti-mouse IgG conjugated to TRITC (1:250, Sigma) or goat-anti-rabbit-IgG conjugated to FITC (1:250, Sigma). Some experiments involved direct staining with IB_4_-FITC conjugates (12.5 μg/ml, Sigma).

The specimens were examined by use of a 20× objective in a fluorescence microscope (Zeiss, Axiovert 200, Germany). The digitized images were captured by AxioVixion software. A total of 1,000 neurons from 8 sections were usually counted, and 95% confidence intervals for proportions were estimated.

### Primary DRG cultures

Mouse DRGs were collected and placed in pre-warmed serum-free DMEM (Invitrogen). After centrifugation at 970 × g for 2 minutes, ganglia were incubated at 37°C for 1.5 hours with 1 ml of serum-free DMEM containing 0.125% collagenase IA (Sigma) and thoroughly mixed in 15-minute intervals, then centrifuged at 970 × g for 3 minutes. Cell pellets were re-suspended in 1 ml of 0.25% trypsin (Invitrogen) and incubated at 37°C for 15 minutes with mixing in 5-minute intervals to avoid aggregation of neurons. After trypsin digestion, cells underwent sedimentation at 1224 × g for 3 minutes and were washed once by DMEM containing 10% fetal bovine serum (FBS), then once with serum-free medium. Ganglia were re-suspended in 2 ml of serum-free DMEM and then dissociated into single cells by mechanical titration 8 times through flame-polished Pasteur pipettes of decreasing tip diameter. Cell suspension was slowly dropped into 10 ml of serum-free DMEM. After 3~5 minutes, the cell suspension on the top (~10 ml) was collected and centrifuged at 1224 × g for 5 minutes. The cell pellet was suspended and mixed in 400 μl DMEM containing 10% FBS and seeded on 100 μg/ml poly-D-lysine-coated 24-mm coverslips. After incubation at 37°C for 2 hours, cells were supplemented with 1.5 ml DMEM containing 10% FBS and maintained at 37°C for 24 hours before use. For cAMP experiments, one-day DRG cultures obtained from CFA-injected mice were treated with the indicated pH HEPES/MES buffers (pH 7.4 and 6.4) for 30 minutes at 37°C, then underwent cAMP assay.

### Constructs and cell cultures

TDAG8 and TRPV1 were cloned into the vector pIRES-hrGFP-2a (pIRES-GFP) for transfection experiments. Human embryonic kidney, adenovirus type 5-transformed 293 cells lines (HEK293T, obtained from Bioresource Collection and Research Center of Food Industry Research and Development Institute, Taiwan) were maintained in DMEM supplemented with 10% FBS (Invitrogen) and antibiotics. For calcium imaging experiments, HEK293T cells were seeded at 4 × 10^5 ^on 24-mm poly-D-lysine-coated coverslips and grown in DMEM containing 10% FBS. Cells were then transfected with 1.5 μg of plasmids pIRES-GFP-TDAG8, pIRES-GFP-TRPV1, or pIRES-GFP by use of lipofectamine reagent (Invitrogen). In some experiments, cells were co-transfected with 1.5 μg of both pIRES-GFP-TDAG8 and pIRES-GFP-TRPV1 in a ratio of 1:1. Intracellular [Ca^2+^] was detected 36 hours after transfection. For cAMP assay, HEK293T cells were seeded at 1.6 × 10^5 ^per well (70%–80% confluence) in 12-well plates. After 24 hours, cells were transfected with pIRES-GFP-TDAG8, pIRES-GFP-TRPV1, or pIRES-GFP plasmids.

### Intracellular calcium imaging

Transfected cells were pre-incubated at 37°C with 2.5 μM Fura-2 acetoxymethyl ester (Fura-2-AM, Molecular Probes) for 40 minutes in a HEPES/MES solution (125 mM NaCl, 1 mM KCl, 5 mM CaCl_2_, 1 mM MgCl_2_, 8 mM glucose, 10 mM HEPES and 15 mM MES, pH7.4). This solution was then replaced with a fresh one without Fura-2-AM. Coverslips were assembled into culture wells and supplemented with 300 μl of the HEPES/MES solution (pH 7.4). Cells were observed by use of a Zeiss inverted microscope and illuminated with a xenon lamp to excitation fluorescence, then images were taken with use of a Zeiss Plan-Apo 63× oil-immersion objective lens. A cooled CCD camera (Photometric) was used to detect fluorescence. GFP-positive cells within a field were identified by use of a FITC filter with excitation wavelength 480 nm and emission wavelength 535 nm. In the same field, fura-2 fluorescence was measured by 10 Hz alternating wavelength time scanning, with excitation wavelength 340 and 380 nm and emission wavelength 510 nm. The fluorescence ratio at two excitation wavelengths (340/380 nm, Ca^2+^-bound Fura-2/free Fura-2) was recorded and analyzed. The HEPES/MES buffers (600 μl) were added to the culture wells to obtain the indicated pH values. The pH-evoked calcium transients and the number of cells responding to the indicated pH values were recorded. Some experiments involved initial supplementation with 500 μl of the HEPES/MES buffer (pH 7.4) in culture wells, then the addition of 500 μl of the HEPES/MES buffer (pH 7.4) containing twice the final agonist concentrations.

### The cAMP assay

Transfected HEK293T cells or primary cultures were pre-incubated for 15 minutes with serum-free DMEM containing 30 μM of the phosphodiesterase inhibitor RO201724 (Sigma), then stimulated with indicated pH buffers containing 30 μM of RO201724 for 30 minutes at 37°C or 22°C. After stimulation, cells were lysed in ethanol. The lysates were dried and cAMP in dried lysates was quantified by use of the cAMP immunoassay kit (Assay Designs, MI), according to the manufacturer's protocol. Some experiments involved pre-incubation with 100 ng/ml of pertussis toxin (PTX) for 2–4 hours or U73122 for 15 minutes at 37°C before pH stimulation. All data are referenced to pH at room temperature. To obtain pH at 37°C, 0.05 pH units should be subtracted for HEPES buffers in the range of pH 6.8–5.0 according to our calibration experiments.

### Statistical analysis

All data are presented as mean ± SEM. Paired *t *test was used to compare the paw volume, withdrawal threshold, and latency between inflammatory agent-treated ipsilateral paws and contralateral paws. The *t *test was used to compare results for control and inflammatory agent-treated groups. The statistically significant levels were set at *p < 0.05, **p < 0.01, and ***p < 0.001.

## Competing interests

The authors declare that they have no competing interests.

## Authors' contributions

YJC participated the study design, performed the experimental procedures and was primary author of the manuscript. CWH performed the primary cultures and calcium imaging experiments. CSL is responsible for the RNA preparation and gene expression experiments. WHC participated in the animal experiments. WHS conceived of the study, and participated in its design, coordination and data interpretation, and contributed to writing the manuscript.
